# Energy Transfer in Metal–Organic Frameworks for Fluorescence Sensing

**DOI:** 10.1021/acsami.1c24759

**Published:** 2022-02-17

**Authors:** Jian-Xin Wang, Jun Yin, Osama Shekhah, Osman M. Bakr, Mohamed Eddaoudi, Omar F. Mohammed

**Affiliations:** † Advanced Membranes and Porous Materials Center, Division of Physical Science and Engineering, King Abdullah University of Science and Technology, Thuwal 23955-6900, Kingdom of Saudi Arabia; ‡ KAUST Catalysis Center, Division of Physical Sciences and Engineering, King Abdullah University of Science and Technology, Thuwal 23955-6900, Kingdom of Saudi Arabia

**Keywords:** energy transfer, luminescent metal−organic frameworks, sensing, photophysics, DFT calculations

## Abstract

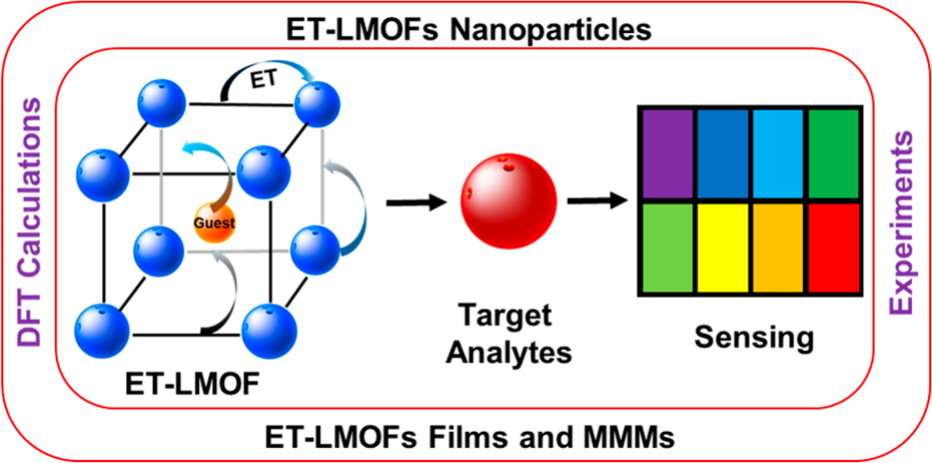

The development of materials with outstanding performance for sensitive and selective detection of multiple analytes is essential for the development of human health and society. Luminescent metal–organic frameworks (LMOFs) have controllable surface and pore sizes and excellent optical properties. Therefore, a variety of LMOF-based sensors with diverse detection functions can be easily designed and applied. Furthermore, the introduction of energy transfer (ET) into LMOFs (ET-LMOFs) could provide a richer design concept and a much more sensitive and accurate sensing performance. In this review, we focus on the recent five years of advances in ET-LMOF-based sensing materials, with an emphasis on photochemical and photophysical mechanisms. We discuss in detail possible energy transfer processes within a MOF structure or between MOFs and guest materials. Finally, the possible sensing applications of the ET-LMOF-based sensors are highlighted.

## 1 Introduction

Metal–organic frameworks (MOFs) are porous-crystalline materials constructed from metal ions or metal clusters coordinated to organic ligands to form one-, two-, or three-dimensional structures, with an outstanding level of structural and compositional control.^1−8^ Introducing emissive organic linkers or metal clusters may lead to the formation of MOF porous structures with interesting luminescent properties. These luminescent metal–organic frameworks (LMOFs)^9−15^ have attracted tremendous attention over the past several years because of their abundant photophysical properties and enormous potential in sensing applications.^11,16−28^ The rational design of LMOFs with energy transfer (ET) characteristics could maximally adjust the luminescent properties of these materials.^29−37^ Because of the outstanding level of structural and compositional control via the flexible selection of organic linkers and metal clusters, energy transfer (ET) LMOFs (ET-LMOFs) are excellent candidates for a variety of potential applications including sensing, photocatalysis, solar cells, X-ray imaging scintillators, and many others.^33,34,38−44^ In addition, the coordination between organic linkers and metal nodes in MOFs allows a high degree of chromophore alignment and organization,^45,46^ which is crucial for the modeling and understanding of the short- and long-distance energy transfer mechanisms.^31,47−51^ The highly ordered and structured periodicity of MOFs also provide an ideal model for density functional theory (DFT) calculations,^52−56^ which is one of the powerful tools for the predetermination of electronic structures and in-depth investigations of the mechanism that underpins the functions of MOFs.

MOFs synthesized via traditional techniques usually lead to the formation of fine powders or tiny particles/crystals with nonthermoplastic properties, insolubility, and difficulty in molding.^57−64^ Thus, processing MOF nanocrystals into specific polymer matrices that are robust and exhibit operational flexibility is highly desired. Such technology not only preserves the individual advantages of MOFs but also overcomes their drawbacks, which greatly expands their practical applications.^10,16,18,65−72^

In this review, we mainly focus on the recent 5 years of development of LMOF crystalline nanoparticles and LMOF-based mixed-matrix membranes (MMMs) with efficient energy transfer processes for sensing multiple analytes. In Section 2, we introduce the mechanisms and strategies for the construction of ET-LMOFs. In Section 3, we discuss the fabrication methods of ET-LMOF-based sensors from experimental and DFT computational perspectives. The sensing applications of ET-LMOF crystalline nanoparticles for different analytes are also discussed in this section. The fabrication and corresponding applications of ET-LMOF films and ET-LMOF MMMs are then reviewed in the last section.

## 2 Luminescent Metal–Organic Frameworks (LMOFs)

**Figure 1:**
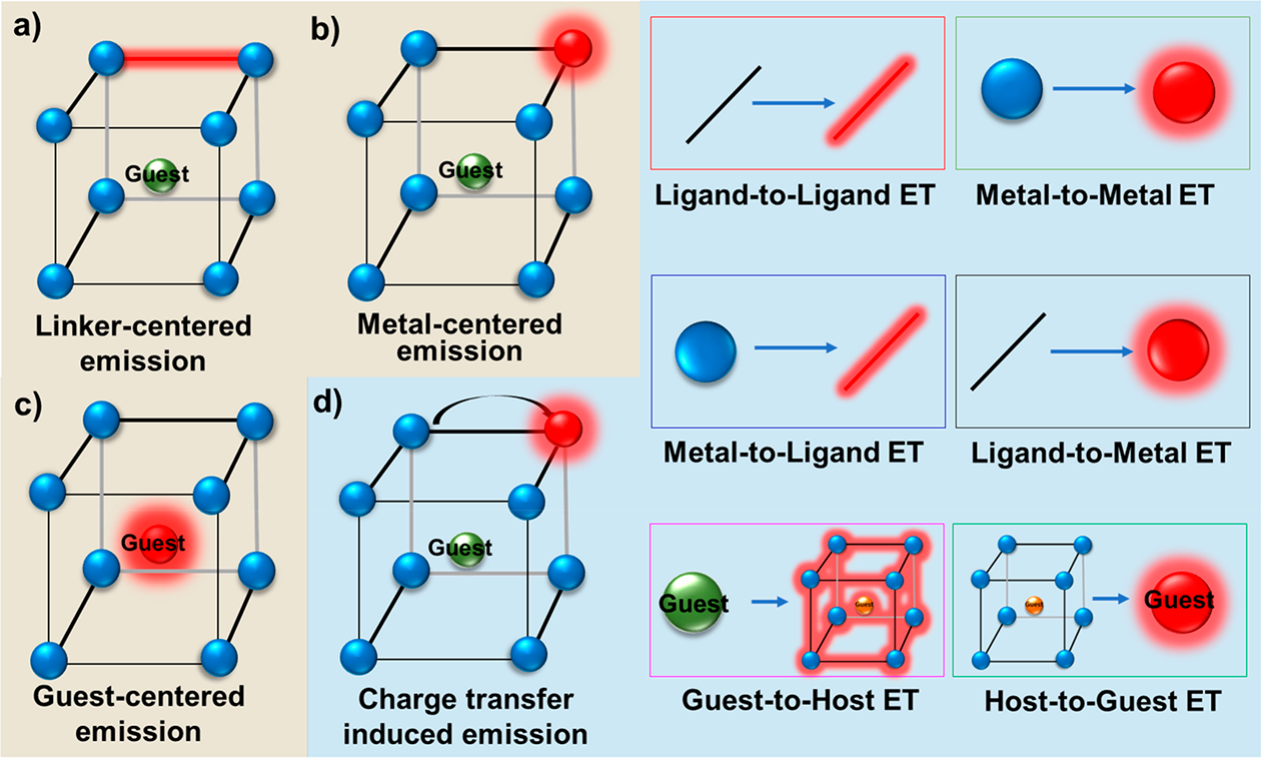
Illustration of the possible luminescent centers and the different energy transfer processes in LMOFs.

MOFs were discovered in the late 1950s, but their enormous applications remained unknown for a long time.^73−87^ Research on MOFs received momentum in the 1990s because of their simple yet intriguing structures and newly discovered properties.^45,88^ For instance, their high porosity and surface areas and excellent structural and functional tunability make them one of the most attractive materials in the chemistry and materials science communities.^24,89−95^ Luminescent metal–organic frameworks are a subclass of MOFs,^11,29,96^ with controllable and tunable photophysical and photochemical properties via alternating the structure of organic linkers, metal clusters, and guest species. The emissions of LMOFs can be roughly divided into four categories, organic linker-centered emission, metal-centered emission, charge transfer induced emission, and guest-centered emission (Figure 1).^96^

### 2.1 Emission Centers in Luminescent Metal–Organic Frameworks

#### 2.1.1 Organic Linker-Centered Emission

**Figure 2:**
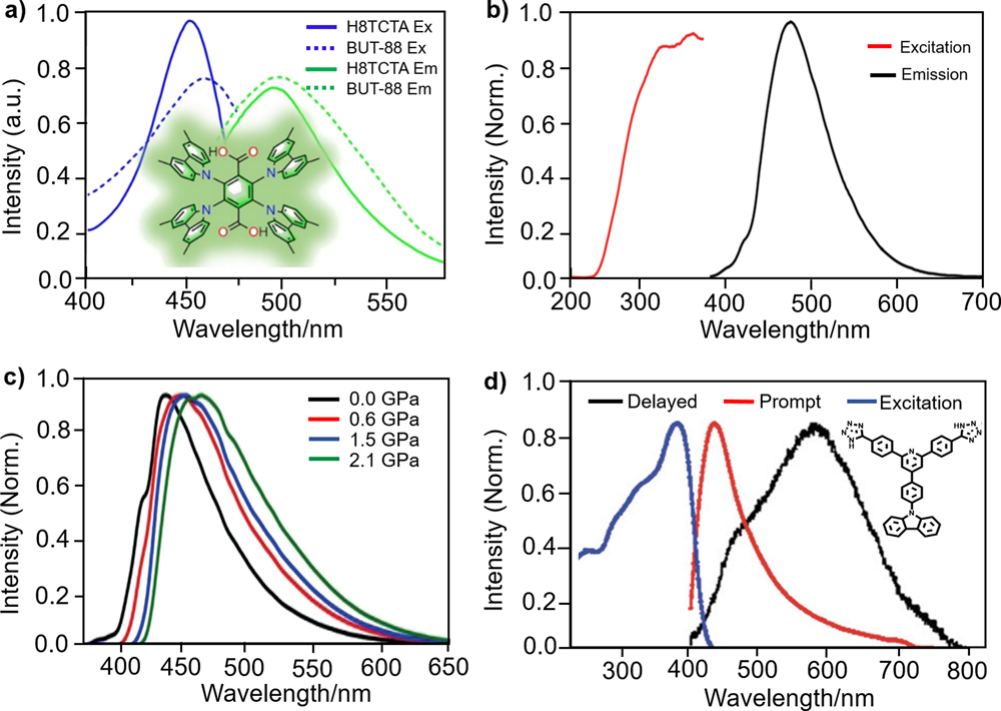
(a) Excitation and emission spectra (450 nm excitation) of the MOF BUT-88 and the structure of the corresponding linker H8TCTA (2,3,5,6-tetrakis(3,6-bis(4-carboxyphenyl)-9H-carbazol-9-yl)-terephthalate). Reprinted with permission from ref (97). Copyright 2020 American Chemical Society. (b) Normalized emission (360 nm excitation) and excitation spectra of NU-1000 (linker: tetraethyl 4,4′,4′′,4′′′-(pyrene-1,3,6,8-tetrayl)tetrabenzoic acid). Reprinted with permission from ref (98). Copyright 2018 The Royal Society of Chemistry. (c) Pressure-dependent emission spectra of the Hf-MOF (linker: 1,4,-phenylene-bis(4-ethynylbenzoate) upon 380 nm excitation. Reprinted with permission from ref (99). Copyright 2020 Wiley. (d) Excitation and prompt and delayed (delayed 0.5 ms) emission spectra of the LIFM-ZCY-1 MOF excited at 365 nm (inset is the molecular structure of the linker). Reprinted with permission from ref (100). Copyright 2020 The Royal Society of Chemistry.

LMOFs with organic linker-centered emission, the extended π-system, or aromatic units are necessary to achieve efficient photoluminescence. It should be noted that anthracene, triazole, naphthalimide, porphyrin, carbazole, perylene, and their derivatives are the most commonly used luminescent linkers.^101−104^ For example, Li and co-workers prepared a linker-based green LMOF, BUT-88, using a carbazolyl derivative (2,3,5,6-tetrakis(3,6-bis(4-carboxyphenyl)-9H-carbazol-9-yl)-terephthalate) as the organic linker. The BUT-88-based probe could selectively and sensitively recognize dual tumor biomarkers (Figure 2a).^97^ A pyrene-based (tetraethyl 4,4′,4′′,4′′′-(pyrene-1,3,6,8-tetrayl)tetrabenzoic acid) LMOF (NU-1000) was applied for accurate sensing of polycyclic aromatic hydrocarbons (PAHs). Because of the efficient preconcentration of 1-hydroxypyrene (1-HP) in NU-1000 and the strong charge transfer interactions between the conjugated 1-HP and pyrene cores, this LMOF exhibited efficient emission quenching-based sensing for 1-HP (Figure 2b).^98^ Moggach and co-workers reported a Hf-LMOF with pressure-induced emission properties,^99^ using 1,4-phenylenebis(4-ethynylbenzoate) as the organic linker. Because of the free rotation properties of the central phenyl ring of the linker, the coplanar arrangement gradually transformed into a twisted configuration under high pressure, leading to the red-shift in both the fluorescence and UV/vis absorption (Figure 2c).^99^ In addition to the development of linker-centered fluorescence LMOFs, linker-centered persistent phosphorescent LMOF for multilevel sensing of oxygen was also reported. The phosphorescent linker was fabricated by connecting an electron donor carbazole (Cz) to an electron acceptor tetrazolyl (Tz). The enhanced intermolecular charge transfer through multiple donor–acceptor, donor−π, and acceptor−π interactions considerably suppressed the nonradiative decay, resulting in efficient linker-centered phosphorescence (Figure 2d).^100^

#### 2.1.2 Metal-Centered Emission

**Figure 3:**
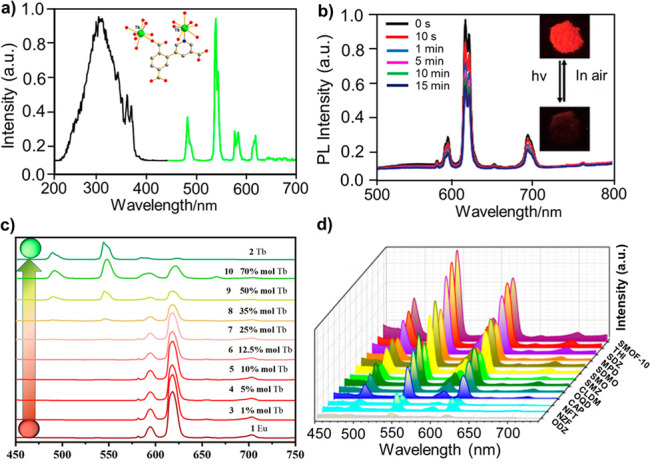
(a) Excitation and emission spectra (320 nm excitation) of the Tb-MOF (linker: 2-(6-carboxypyridin-3-yl)terephthalic acid) and the coordination environment of the Tb1 and Tb2 centers. Reprinted with permission from ref (105). Copyright 2018 The American Chemical Society. (b) Fluorescence spectra of the Eu-MOF (linker: 1-(3,5-dicarboxybenzyl)-10-(R-2,3-dihydroxypropyl)-4,40-bipyridinium dichloride) at different irradiation times under 395 nm excitation. Reprinted with permission from ref (106). Copyright 2020 The Royal Society of Chemistry. (c) Emission spectra of Eu_1–*x*_CTb_*x*_-MOF (linker: (5,5′-(propane-1,3-diylbis(oxy))di-isophthalic acid) at 330 nm excitation and (d) the fluorescence response of the membrane containing Eu_0.3_Tb_0.7_-MOF toward different antibiotics. Reprinted with permission from ref (107). Copyright 2020 The American Chemical Society.

Ln^3+^ ions are commonly used in LMOFs with metal-centered emission. However, photoluminescence quantum yield (PLQY) of Ln^3+^ is low because of the forbidden transitions. In some MOFs, the low PLQY can be improved by the antenna effect of appropriate organic linkers. Liu’s group reported a series of Ln^3+^-based LMOFs, using nitrates and 2-(6-carboxypyridin-3-yl)terephthalic acid as the linkers.^105^ Both of these LMOFs exhibited efficient Ln^3+^ metal-centered emission with high selectivity toward Ce^3+^ or Fe^3+^ ions (Figure 3a). Being in this regime, Zang’s group developed a novel viologen-based two-dimensional luminescent Eu-MOF.^106^ Because of the characteristic emission of Eu^3+^ and the electron-deficient properties of the viologen linker (1-(3,5-dicarboxybenzyl)-10-(R-2,3-dihydroxypropyl)-4,40-bipyridinium dichloride), this Eu-MOF exhibited excellent sensitivity toward light (Figure 3b). To further enhance the optical properties, Zhao and co-workers constructed bimetallic lanthanide MOFs Eu_1–*x*_Tb_*x*_-MOF, using (5,5′-(propane-1,3-diylbis(oxy))di-isophthalic acid as the organic linker.^107^ The constructed mixed matrix membrane with the Eu_0.3_Tb_0.7_-MOF exhibited clear emission from both Eu^3+^ and Tb^3+^ ions and could thus be used as ratiometric self-calibrating luminescent probes for the sensing of various antibiotics (Figure 3c, d).

#### 2.1.3 Charge Transfer Induced Emission

The electrons in LMOFs are not always localized on the linker or metal centers, but they can be delocalized across the whole framework. Therefore, charge transfer processes such as linker-to-linker charge transfer (LLCT), linker-to-metal charge transfer (LMCT), metal-to-linker charge transfer (MLCT), and metal-to-metal charge transfer (MMCT) could be involved. Among them, LMCT is generally observed in Zn^2+^ and Cd^2+^ compounds, whereas MLCT can be observed in Cu^+^ and Ag^+^ compounds.^110^ In addition, these mechanisms generally do not exist independently, but two or more processes coexist.^111−114^

**Figure 4:**
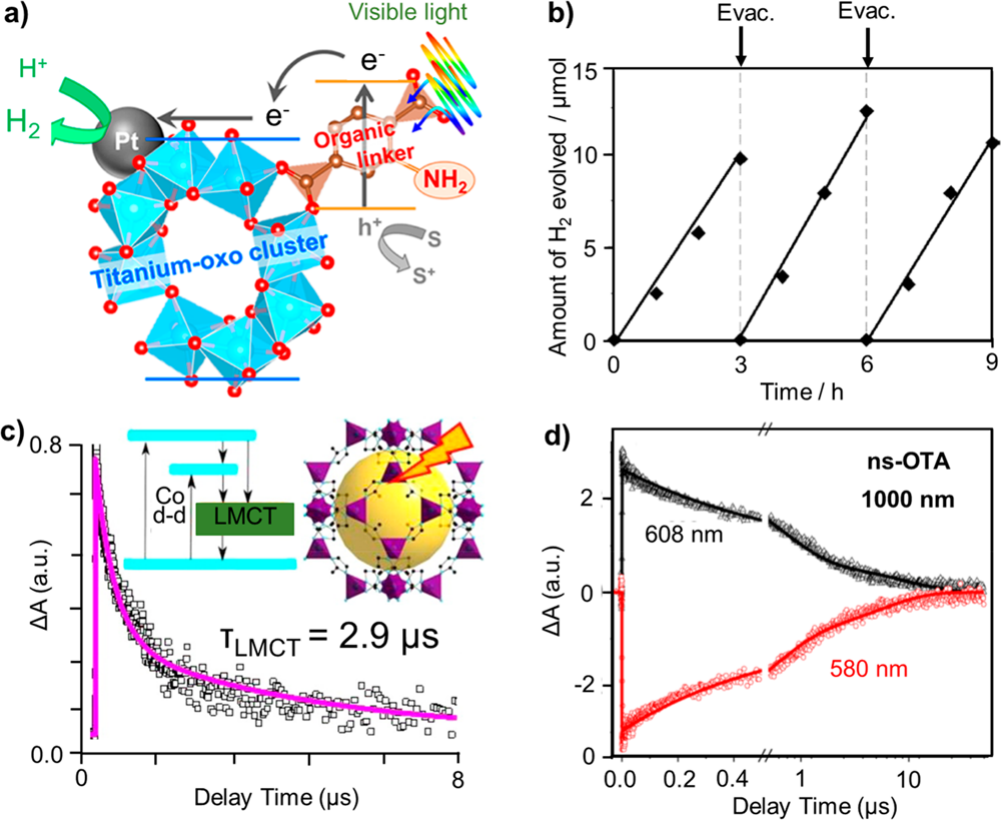
(a) Amino-functionalized Ti-MOF (linker: 2-amino-benzenedicarboxylic acid) used for the photocatalytic production of hydrogen, (b) time course of photocatalytic hydrogen production under visible-light irradiation (λ > 420 nm). Reprinted with permission from ref (108). Copyright 2012 American Chemical Society. (c) Emission decay profile and excited-state relaxation diagram of ZIF-67 (linker: 2-methylimidazole), (d) the kinetics at 608 and 580 nm in nanosecond optical transit absorption (ns-OTA) spectra of ZIF-67 upon 1000 nm excitation. Reprinted with permission from ref (109). Copyright 2016 American Chemical Society.

Matsuoka and co-workers reported an amino-functionalized Ti^4+^ MOF (TiMOF-NH_2_), with efficient photocatalytic performance in hydrogen production.^108^ It was found that the photocatalytic property resulted from photoinduced electron transfer from triethanolamine to the catalytically active titanium-oxo cluster. The photoinduced charge transfer within the organic linker (2-amino-benzenedicarboxylic acid) also boosted its photocatalytic performance (Figure 4a and 4b). The long-lived charge-separated excited state in a ZIF (ZIF-67) with good photocatalytic properties was observed by Huang and co-workers.^109^ According to the optical transient absorption (OTA) and X-ray transient absorption (XTA) measurements upon the photoexcitation of the spin allowed d-d transition of Co^2+^ ion in ZIF-67, the long-lived intermediate state within a picosecond time scale was clearly demonstrated. The long-lived charge-separated state and intrinsic hybrid nature endow these materials with high potentials for heterogeneous photocatalysis and energy conversion (Figure 4c, d).

#### 2.1.4 Guest-Centered Emission

Due to the highly ordered structures and tunable pore size of LMOFs, different kinds of luminescent guests, such as quantum dots, metal clusters, and organic dyes can be encapsulated.^117,118^ The porosity of LMOFs could also be used to constrain the analyte–MOF distances, which greatly enhance the interactions between the MOFs and analytes.^11^ The unique adsorption properties of LMOFs make the analytes be preconcentrated and drastically improve their sensing performance.^11^

**Figure 5:**
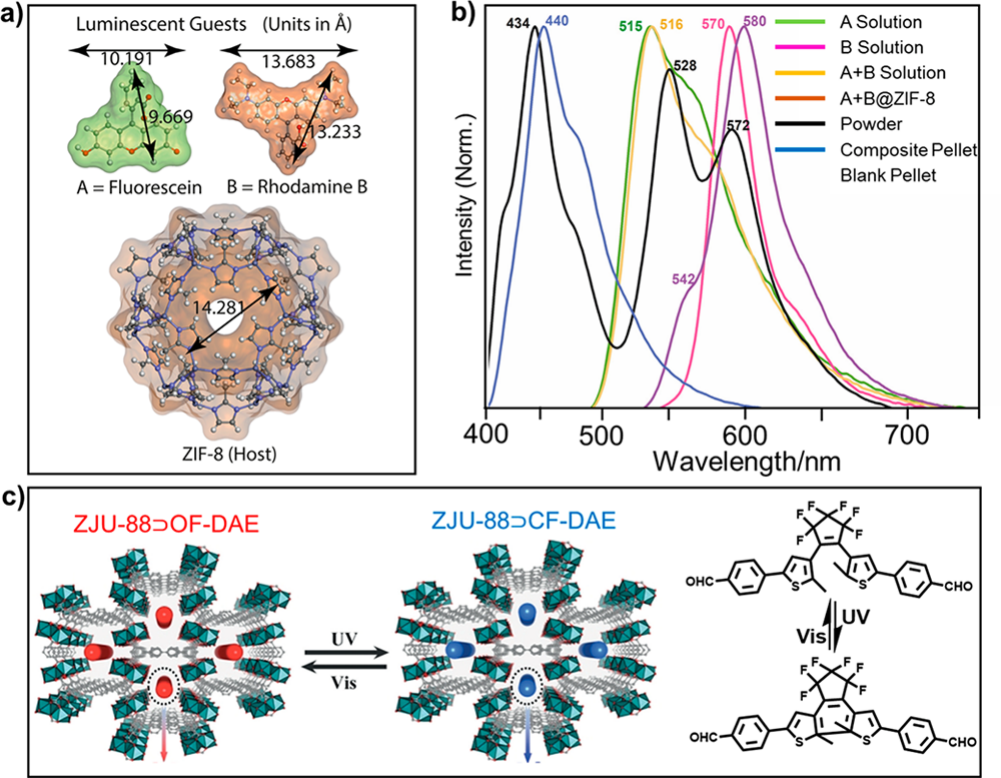
(a) Structural dimensions of A, B, and ZIF-8; and (b) corresponding emission spectra upon 400 nm excitation. Reprinted with permission from ref (115). Copyright 2020 Wiley. (c) Photochromic properties of ZJU-88⊃OF-DAE (linker: 1,1′:4′,1′′:4′′,1′′′-quaterphenyl-3,3′′′,5,5′′′ tetracarboxylic acid) under different excitation wavelength. Reprinted with permission from ref (116). Copyright 2019 Wiley.

Recently, Tan and co-workers encapsulated green fluorescein and red rhodamine B into ZIF-8 and combined this composite with a blue-emitting photopolymer resin to achieve color-tunable 2D printable luminescent composite materials (Figure 5a, b).^115^ Chen and co-workers introduced a diarylethene photoswitch into a lanthanide MOF, ZJU-88, using 1,1′,4′,1′′,4′′,1′′′-quaterphenyl-3,3′′′,5,5′′′ tetracarboxylic acid as the organic linker.^116^ The energy transfer between the MOF and diarylethene can be reversibly regulated by light-triggered open-closed forms of the diarylethene unit. Because of the photoswitchable luminescent properties, this composite has been used successfully in information anticounterfeiting (Figure 5c).

### 2.2 Energy Transfer Mechanism in Luminescent Metal–Organic Frameworks

Energy transfer can be simply understood as the transfer of excited-state energy from the donor to a neighboring ground-state acceptor. It results in simultaneous deactivation and excitation of the energy donor and acceptor, respectively, as described in eq 1.
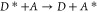
1In general, nonradiative energy transfer mechanisms can be divided into two main categories: Förster and Dexter energy transfer mechanisms.

Förster resonance energy transfer (FRET) is a short-range^119^ energy transfer mechanism, in which the excitation energy is transferred to the energy acceptor in a nonradiative fashion. Because of the weak dipole–dipole interactions, the rate of FRET (*k*_FRET_) depends on the inverse sixth power of the separation distance between the donor and the acceptor. It should be noted that FRET requires strong spectral overlap between the donor’s emission and the acceptor’s absorption spectra to ensure the thermodynamic feasibility of energy transfer.

Dexter energy transfer relies heavily on the orbital overlap between donor and acceptor. The electron exchange mechanism can be described as an intuitive two-step electron transfer that effectively exchanges electronic excitation. The rate of the Dexter energy transfer depends exponentially on the distance between the donor and the acceptor (*R*) and increases exponentially as steeply as the rate of hypothetical electron transfer between the same molecules with a decrease in *R*.

The profound understanding of FRET and Dexter energy transfer mechanisms provide a theoretical basis for the singlet–singlet or triplet–triplet energy transfer in LMOFs from linker to linker, linker to metal center, or LMOFs to guest molecules, enabling researchers to design different energy transfer systems that greatly enrich the development of LMOFs.

### 2.3 Energy Transfer Processes in Luminescent Metal–Organic Frameworks

LMOFs coupled with energy transfer provide additional ratiometric detection modes for different analytes.^29,30,120^ In this section, we will summarize linker-to-linker, linker-to-metal, metal-to-metal, and host–guest energy transfer in LMOFs and their corresponding sensing applications.

#### 2.3.1 Linker-to-Linker Energy Transfer

**Figure 6:**
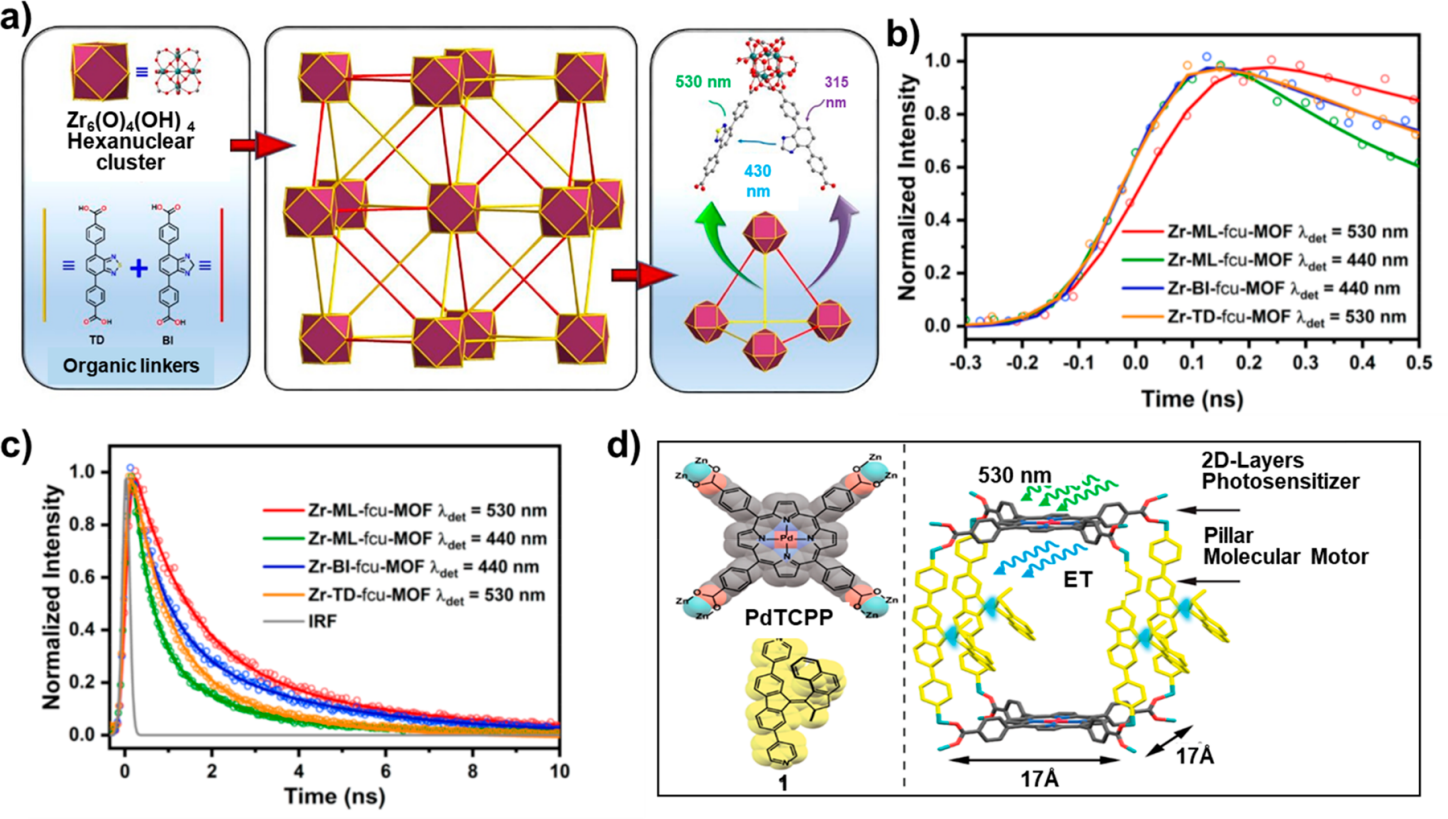
(a) Illustration of the linker-to-linker energy transfer in mixed ligands Zr-ML-**fcu**-MOF. TCSPC data for the Zr–BI-**fcu**-MOF (BI: 4,4′-(1H-benzo[d]imidazole-4,7-diyl)dibenzoic acid), Zr-TD-**fcu**-MOF (TD: 4,4′-(benzo[c][1,2,5]-thiadiazole-4,7-diyl)dibenzoic acid), and Zr-ML-**fcu**-MOF at their emission maxima in acetonitrile solution; (b) on an early time scale and (c) on a long time scale upon 350 nm excitation. Reprinted with permission from ref (121). Copyright 2020 American Chemical Society. (d) Visible-light-driven rotation of molecular motors in a dual-function MOF enabled by linker-to-linker energy transfer. Reprinted with permission from ref (122). Copyright 2020 American Chemical Society.

Linker-to-linker energy transfer can be achieved by coupling both the energy donor and acceptor within the same MOF structure. The energy transfer processes in these systems can be controlled by external stimuli, such as the excitation wavelength, temperature, or pH. This concept was recently realized by Mohammed’s and Eddaoudi’s groups. In this work, the efficient linker-to-linker energy transfer (approximately 90% efficiency) from the benzimidazole (energy donor) to the benzothiadiazole (energy acceptor)-functionalized linkers within one MOF structure was observed. The similar molecular structural features of the donor and acceptor enabled the successful synthesis of the colinker MOF with a large degree of spectral overlap (Figure 6a).^121^ The authors also estimated experimentally the donor–acceptor distance within the MOF framework, which is in good agreement with the calculated values for neighboring ligands inside Zr-**fcu**-MOFs (Figure 6b, c). Saha and co-workers prepared a pillared paddle wheel LMOF by coordinating Zn^2+^ ions with the mixed linker of naphthalene dicarboxylate (NDC) and *N,N*′-di(4-pyridyl)thiazolo-[5,4-*d*]thiazole (DPTTZ). Because of the highly ordered arrangement between the donor (NDC) and acceptor (DPTTZ), and the good spectral overlap, a high energy transfer efficiency was achieved. In addition, this ET-LMOF exhibited high sensitivity and selectivity toward Hg^2+^.^123^

Photochromic materials exhibit reversible color transformation under photoirradiation and possess high potentials in data storage, erasable and inkless printing, optical transmission materials, etc. However, most traditional classes of such materials are based on the reversible structural interconversion of spiropyran, diarylethene, azobenzene, and redox-active cores.^12^ These strategies always show a fast discoloration process, and it is difficult to integrate them into specific physical forms for practical applications. Feringa and co-workers inserted a visible-light-driven molecular motor into a dual-function MOF and realized efficient energy transfer between different linkers. They selected a palladium-porphyrin photosensitizer (palladium-porphyrin tetracarboxylic acid) and an overcrowded alkene-based molecular motor (bispyridine-derived linker 1) as the linkers (energy donor and acceptor, respectively). The efficient triplet–triplet energy transfer from the porphyrin linker to the molecular motor makes the framework capable of harvesting low-energy green light to power the rotary motion of the molecular motor (Figure 6d).^122^

#### 2.3.2 Linker-to-Metal Energy Transfer

In linker-to-metal energy transfer LMOFs, after the organic linker is optically excited, the excitation energy is quickly transferred to metal ions and then emit from the metal center. Murugesu and co-workers reported a class of three-dimensional Ln LMOFs by using H_2_NDC (2,6-naphthalenedicarboxylate) as the organic linker. An in-depth theoretical and experimental analysis demonstrated that the organic linker acted as the antenna (energy donor) and that Ln^3+^ acted as the emission-center (energy acceptor). The linkers harvest the excitation energy and then transfer it to the emitting levels of Ln^3+^ ions, leading to the enhanced photoluminescence.^41^ With a similar mechanism, Spodine and co-workers successfully improved the Tb^3+^-centered luminescence by tuning the linker-to-metal energy transfer efficiency. The authors tuned the energy gap between the triplet state of the linker (2,2′-[[(2-pyridinylmethyl)-imino]di(methylene)]-bis(4-R-phenol)) and the emissive ^5^D_4_ level of Tb^3+^ by regulating the substituents on the linker. Finally, the linker’s emission could be quenched completely, indicating efficient energy transfer from the linker to the metal center.^112^

#### 2.3.3 Metal-to-Metal Energy Transfer

Metal-to-metal energy transfer in LMOFs is usually used in noninvasive luminescent thermometers with a broad working temperature range. In 2010, Lin and co-workers systematically investigated the energy transfer from Ru to Os in isomorphous MOFs. They studied the energy transfer dynamics by using two-photon excitation methods. A decrease in the emission lifetime at 620 nm of Ru from 171 to 29 ns was observed as the doping ratio of Os increased to 2.6 mol %, while the emission intensity of Os increased at the same time. Both of these results indicate rapid and efficient energy transfer from Ru to Os ions in these isomorphous MOFs.^124^ Energy transfer from Tb^3+^ to Eu^3+^ is another important and well-studied metal-to-metal energy transfer system that has been widely used in the design of ratiometric thermometers.^125−128^ For example, Jin and co-workers systematically investigated the energy transfer from Tb^3+^ to Eu^3+^ in a mixed MOF by using 2,5-dimethoxy-1,4-benzenedicarboxylic acid as the organic linker. The energy transfer dynamics were studied in detail by using time-resolved spectroscopy. In addition, the energy transfer efficiency and the emission color could be finely tuned by adjusting the ratio of Tb^3+^ to Eu^3+^.^125^

#### 2.3.4 Luminescent Metal–Organic Frameworks- Guests Energy Transfer

**Figure 7:**
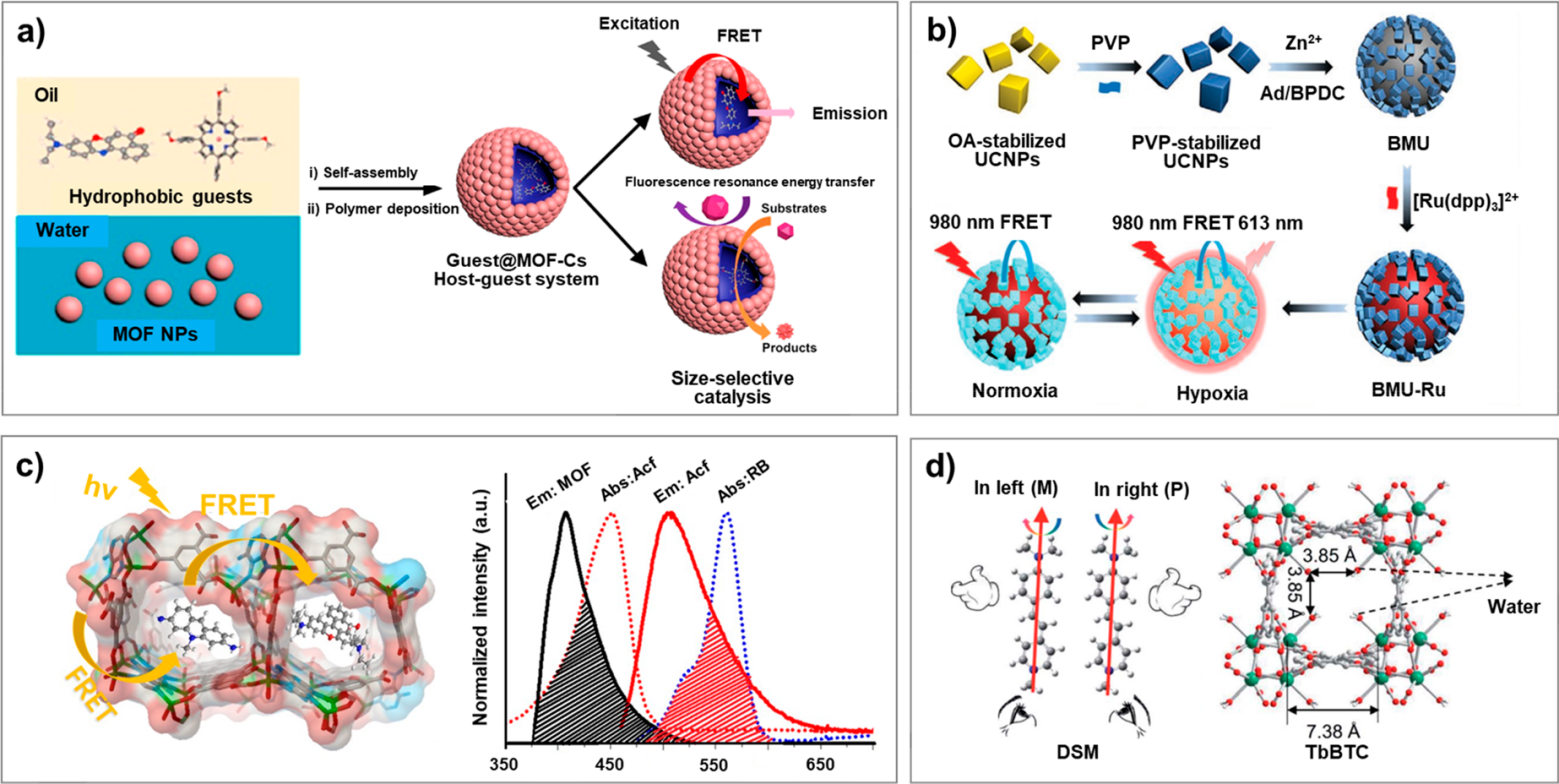
(a) Effective method for the encapsulation of hydrophobic guests inside MOF capsules (MOF-C-s). Reprinted with permission from ref (129). Copyright 2020 American Chemical Society. (b) Fabrication of an MOF-based sensor for hypoxia detection. Reprinted with permission from ref (34). Copyright 2020 Wiley. (c) Tandem FRET pathway in a mixed-dye@ZnBTCA system and the spectra overlap between the emission spectrum of ZnBTCA (Zn_3_(benzene-1,3,5-tricarboxyl)_2_(adenine)(H_2_O)) and the absorption spectrum of Acf (biological stain acriflavine). Reprinted with permission from ref (40). Copyright 2019 Wiley. (d) Chiral MOF system achieved by energy transfer strategy. Reprinted with permission from ref (35). Copyright 2020 The Royal Society of Chemistry.

Because of the tunable topology of porous frameworks, LMOFs can encapsulate various desired guest molecules, offering a unique opportunity to investigate the host–guest energy transfer mechanisms and their sensing performance.^12,19^ Because the guest molecules can serve as a simple luminescence probe or as the analyte, the LMOF-guest is promising system for sensing applications. Recently, Zhang and co-workers proposed a strategy for effectively encapsulating hydrophobic guests inside MOF capsules (MOF-Cs). They synthesized the MOF-Cs by the self-assembly of surface-modified MOF nanoparticles (UiO-66-NH_2_) at the water–oil interface to form oil-in-water emulsions, and then poly(methyl methacrylate) (PMMA) was deposited into the emulsions by an internal phase separation method (Figure 7a). The MOF-Cs can encapsulate various types of hydrophobic guests, which improve energy transfer and promote size-selective catalysis.^129^ Another interesting near-infrared (NIR)-excited nanosensor was constructed by Tang and co-workers by using a biological MOF (bio-MOF-100, linker: 4,4′-biphenyldicarboxylic acid) as a matrix to encapsulate a donor (rare-earth-doped upconversion nanoparticles (UCNPs))-acceptor ([Ru(dpp)_3_]^2+^Cl_2_: tris(4,7-diphenyl-1,10-phenanthroline) ruthenium(II) dichloride) energy transfer system. Under NIR excitation, the core/satellite nanosensors exhibit enhanced FRET efficiency and high sensitivity toward hypoxic atmosphere (Figure 7b).^34^ By adjusting the FRET efficiency from LMOFs to organic dyes, Li and co-workers constructed a series of high-performance luminescent ratiometric thermometers. A biological MOF (ZnBTCA: Zn_3_(benzene-1,3,5-tricarboxyl)_2_(adenine)(H_2_O)) was selected as the host material, and a thermosensitive organic fluorophore (biological stain acriflavine) was used as the acceptor to establish a temperature-tunable FRET system (Figure 7c), in which the energy transfer efficiency can be fine-tuned by temperature variation.^40^ In addition to singlet-to-singlet energy transfer, the triplet-to-singlet energy transfer between MOFs and guest molecules was also well studied. Such system was fabricated by encapsulating a series of fluorescent dyes into the green phosphorescent MOF Cd(1,3-benzenedicarboxylic acid)(benzimidazole). Due to the slow energy transfer from the MOFs to the dyes, a series of stable persistent LMOFs with different emission colors were obtained. These composites exhibit high potentials in data anticounterfeiting.^130^ Moreover, Yao and co-workers developed an efficient energy and circularly polarized luminescence (CPL) transfer system. They encapsulated an achiral stilbazolium dye (DSM: (4-*p*-(dimethylamino)styryl)-1-methylpyridinium iodide) into homochiral lanthanide MOFs (TbBTC), in which the P- or M-configuration of the dye is unidirectionally generated via spatial confinement of the MOF and solidified by the dangling water molecules in the channel. By tuning the ratio between the donor and acceptor, a series of color-tunable CPL luminescent materials were successfully fabricated (Figure 7d).^35^

## 3 Sensing Applications of Luminescent Metal–Organic Frameworks

### 3.1 Density Functional Theory (DFT) Calculations in Luminescent Metal–Organic Frameworks

**Figure 8:**
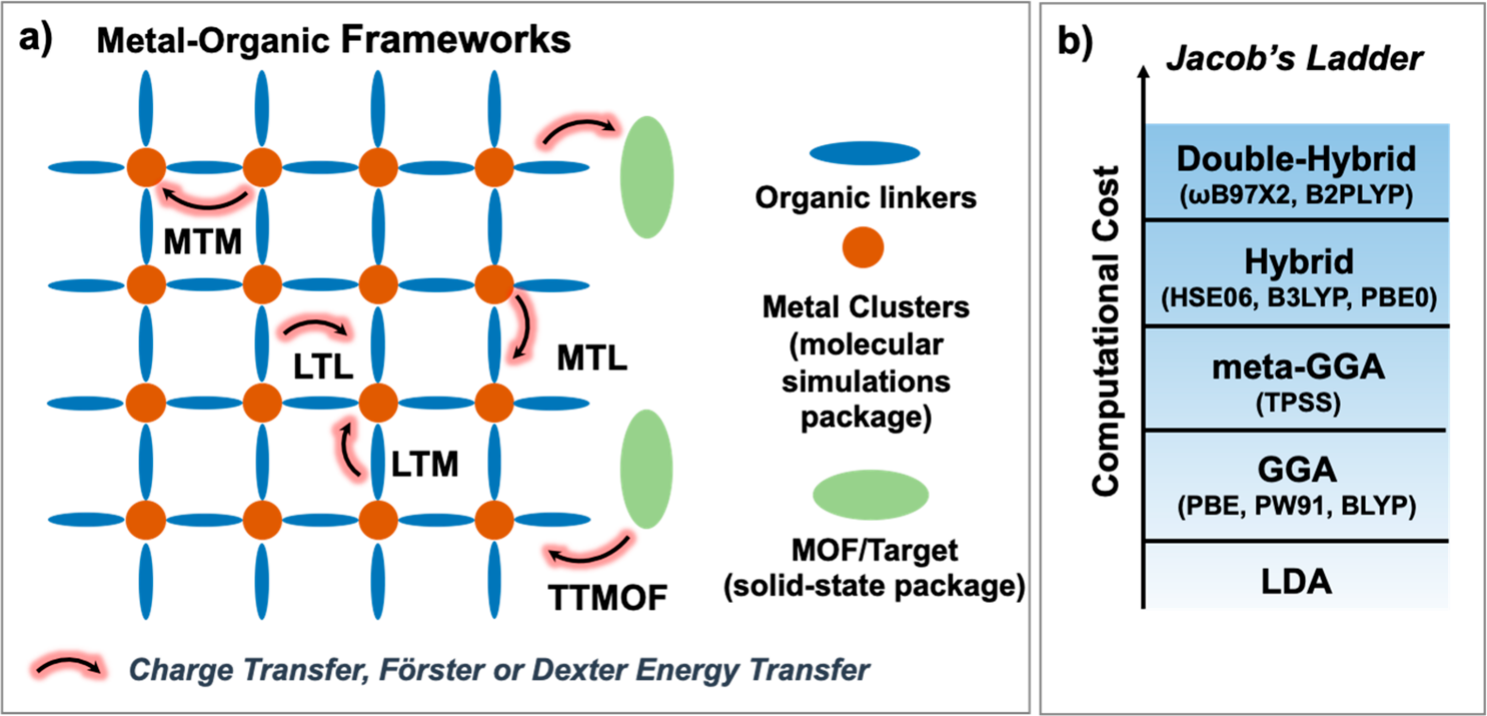
(a) Schematic illustration of energy transfer within MOFs and between MOFs and guests: organic linkers or metal clusters can be treated as molecules, and MOFs/guests can be treated as extended solids; (b) Jacob’s ladder for the functional used in the density functional theory (DFT) calculations.

Density functional theory (DFT) calculations have become a powerful theoretical tool for investigating the structural, electronic, and optical properties of MOFs because of the development of advanced DFT functions and the widespread availability of high-performance computing resources. In particular, DFT calculations can be used to study the interactions and the mechanism underlying the charge and energy transfer between organic linkers and metal clusters in MOFs and between MOFs and targets (Figure 8a). For a specific MOF structure or MOF/target system, it is critical to identify which DFT method provides the best electron density or energy based on the parameters that are considered in constructing the function.^131,132^ However, no functions can fully satisfy the Hohenberg and Kohn theorem, so it is hard to obtain the exact electron density for the ground state. To overcome this, different functions have been developed to treat the exchange-correlation term. Figure 8b shows a Jacob’s ladder diagram, in which exchange and correlation functions with the same capabilities are categorized on the same rung. The local density approximation (LDA) is located on the lowest rung and is solely based on the electron density (e.g., the parametrizations developed by Perdew and Zunger).^133^ The second-lowest rung shows the generalized gradient approximation (GGA) that takes into account the gradient of the electron density, such as the parametrizations developed by Perdew, Burke, and Ernzerhof (PBE);^134^ the revised PBE for solids (PBEsol);^135^ and PW91.^136^ The next rung contains meta-GGAs that include the second derivative of the electron density and the orbital kinetic energy density, such as TPSS.^137^

Hybrid functions replace a predefined fraction of the underlying exchange and correlation energy with a Hartree–Fock exact exchange term, such as the parametrizations developed by Heyd, Scuseria, and Ernzerhof (HSE),^138^ PBE0,^139^ and B3LYP.^140^ These hybrid functions have been commonly used for modeling MOFs. By climbing Jacob’s ladder and applying more sophisticated exchange and correlation functions, one may expect to obtain improved accuracy from the DFT calculations. However, this is always accompanied by an increased computational cost, and hence, a compromise between accuracy and computational cost should be carefully considered in the DFT calculations. In the following, we review the recent DFT studies on molecular adsorption, the interactions between MOFs and guests, and the charge and ET in MOF-based structures.

Employing the standard DFT functional with van der Waals (vdW) corrections is imperative to exploring the adsorption of molecules and other materials on MOFs. For example, Rosen et al. investigated the O_2_ and N_2_ adsorption properties in several coordinatively unsaturated MOFs based on DFT calculations (at the PBE level with D3(BJ) corrections).^54^ They demonstrated the possibility of tuning O_2_ affinity at the open metal sites of MOFs and found that the O_2_ affinity was much stronger than the N_2_ adsorption. They also found that replacing the anion exchange of bridging ligands μ-Cl^–^ of M_2_Cl_2_(BBTA) with μ-OH^–^ significantly enhanced the O_2_ adsorption ability but not the N_2_ affinity. Chibani and co-workers studied the adsorption behavior of RuO_4_ onto MOFs and zeolites to mitigate ruthenium release from the porous matrix by using DFT calculations (at the PBE level with DFT-D3 corrections).^141^ They found that the nature of the porous materials had an inconspicuous effect on the adsorption energy of RuO_4_, but the generation of hydrogen bonds between the H atoms of water molecules and the O atoms of RuO_4_ made a major contribution.^142^

**Figure 9:**
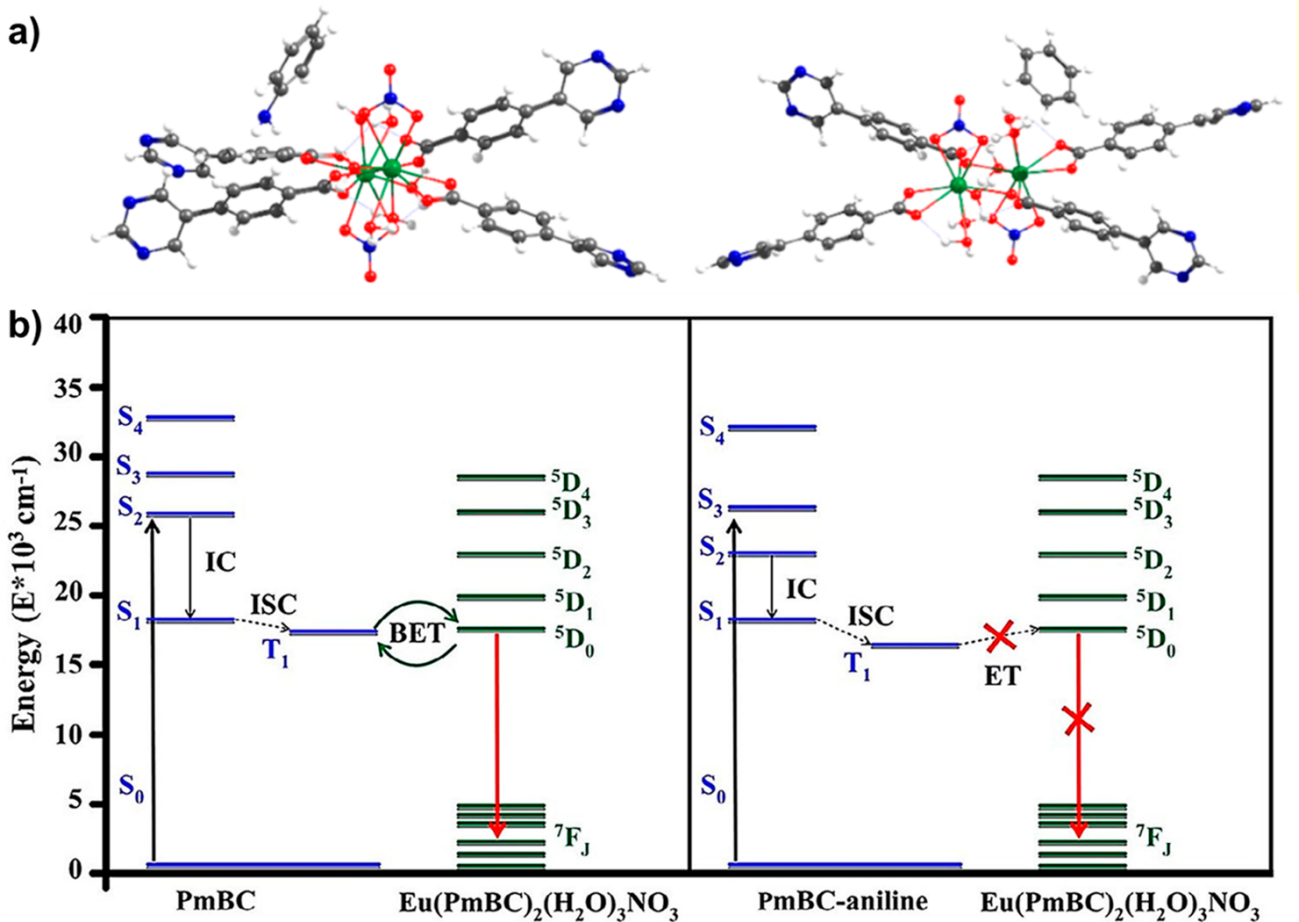
(a) Optimized geometries of the Eu-MOF-aniline (left) and Eu-MOF-benzene (right), (b) energy transfer pathway for the sensitization and emission of the Eu-MOF system. Reprinted from ref (143). Copyright 2020 Wiley.

Revealing the charge and energy transfer mechanisms between organic linkers and metal clusters in MOFs and between MOFs and targets strongly relies on DFT calculations. Hidalgo-Rosa et al. investigated the detection mechanism of an Eu-based MOF toward aniline by using simplified time-dependent DFT (sTD-DFT) calculations (at the GGA/BP86 and CAM-B3LYP levels of theory).^143^ They showed that energy was transferred from the linker’s triplet excited state to the ^5^D_0_ state of Eu^3+^ and then emitted upon transition from the ^5^D_0_ to the ^7^F_J_ state. Moreover, a mixture of electronic states between the Eu-based MOF and aniline was observed, making the molecular orbitals of aniline appear in the active space that stabilizes the linker and block the energy transfer to the ^5^D_0_ state of Eu^3^ (Figure 9). An efficient energy transfer between a Zr-**fcu**-BADC-MOF (BADC: [9,9′-bianthracene]-10,10′-dicarboxylic acid) and an organic chromophore was recently reported.^144^ In this MOF-chromophore structure, a short intercomponent distance of 10.9 Å was estimated, resulting in a nearly 100% energy transfer efficiency. In addition, the type-1 energy alignments between the MOF and the organic chromophore also suggest feasible energy transfer from the MOF to the organic chromophore.^144^

### 3.2 Fabrication of Luminescent Metal–Organic Framework-Based Sensors

**Figure 10:**
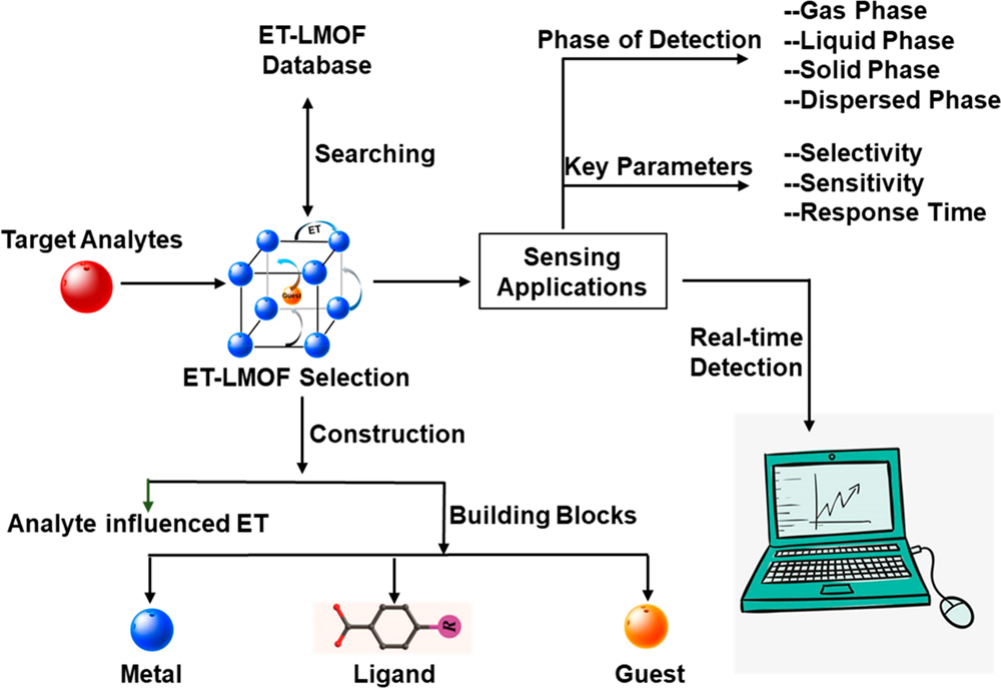
Schematic outline for the design of the ET-LMOF-based sensors.

As mentioned above, the designable permanent porosity of MOFs endows them with reliable and accurate selectivity. The well-defined shape, size, and porous channels provide practical methods for the investigation of adsorption kinetics and identification of the response time. The building units and their corresponding assembly make it easy to fabricate a target-specific system, which allows MOFs to function as communication devices for a variety of analytes. Luminescence is a powerful signal used for the detection of a variety of analytes.^11^ The luminescence intensity, decay profile, and emission wavelength can be used alone or in combination to recognize the presence of interacting species. Additionally, introducing energy transfer into LMOFs greatly enhances their luminescent properties^11,29^ and leads to highly sensitive recognition of the detected analytes by regulating the energy transfer between the LMOFs and analytes (Figure 10).^145^

A single change in intensity is not a precise method for recognition because it can be influenced by the surroundings, such as the sample conditions, solvents, temperature, or even the excitation source.^146−149^ The ratiometric changes of two or more emissions in one sensor exhibit higher sensitivity. To prepare these types of ratiometric sensors, we can design LMOFs with multiple luminescent linkers or introduce linker-to-linker or metal-to-metal energy transfer into the systems.^150,151^ Therefore, once the analytes show different influences on different emission centers or regulate the energy transfer process, the analytes can be accurately detected by using the change in the ratio of the multiple emissions. Additionally, the choice of metal ions is a very important factor. For example, paramagnetic transition metals such as Mn^2+^, Co^2+^, and Cu^2+^ are typically emission quenchers because of the promoted nonradiative excited-state decay. On the other hand, some d^10^ systems, such as Cu^+^, Ag^+^, Cd^2+^, and Zn^2+^, always show good emission properties because of the enhanced radiative decay by the MLCT or LMCT effect.^96^

Moreover, stability is another significant parameter that must be considered carefully in the process of LMOF selection to obtain high-efficiency ET-LMOF-based sensors. Most of the reported MOFs show bad stability in moisture/aqueous environments because of the inevitable decomposition of the metal-linker bond. To date, several works have been conducted to investigate the stability of MOFs to broaden their applications in real-time detection. For example, coordinating high-valent metal ions (Zr^4+^, Cr^3+^, Al^3+^, Fe^3+^) with O donor linkers (hard bases) would greatly enhance the chemical stability.^152−154^ The high-valent metal ions not only provide high charge density (hard acids) but also lead to a high coordination number of the metal center and further enhance its stability. It should be noted that if low-valent metal ions (such as Co^2+^, Ni^2+^, Fe^2+^, and Ag^+^) need to be used as the metal center, a suitable N-containing linker could enhance the basic and moisture stability of the MOFs.^155^

### 3.3 Sensing Applications of Luminescent Metal–Organic Frameworks

ET-LMOFs exhibit unique characteristics for selective capture of analytes and have been widely used in sensors design.^29^ Because of the permanent porosity of MOFs, reversible adsorption and release of analytes can be easily realized. Therefore, ET-LMOFs were demonstrated with high potentials in the sensing of organic molecules, nanoparticles, biomarkers, and explosive-like molecules.^11,29^

**Figure 11:**
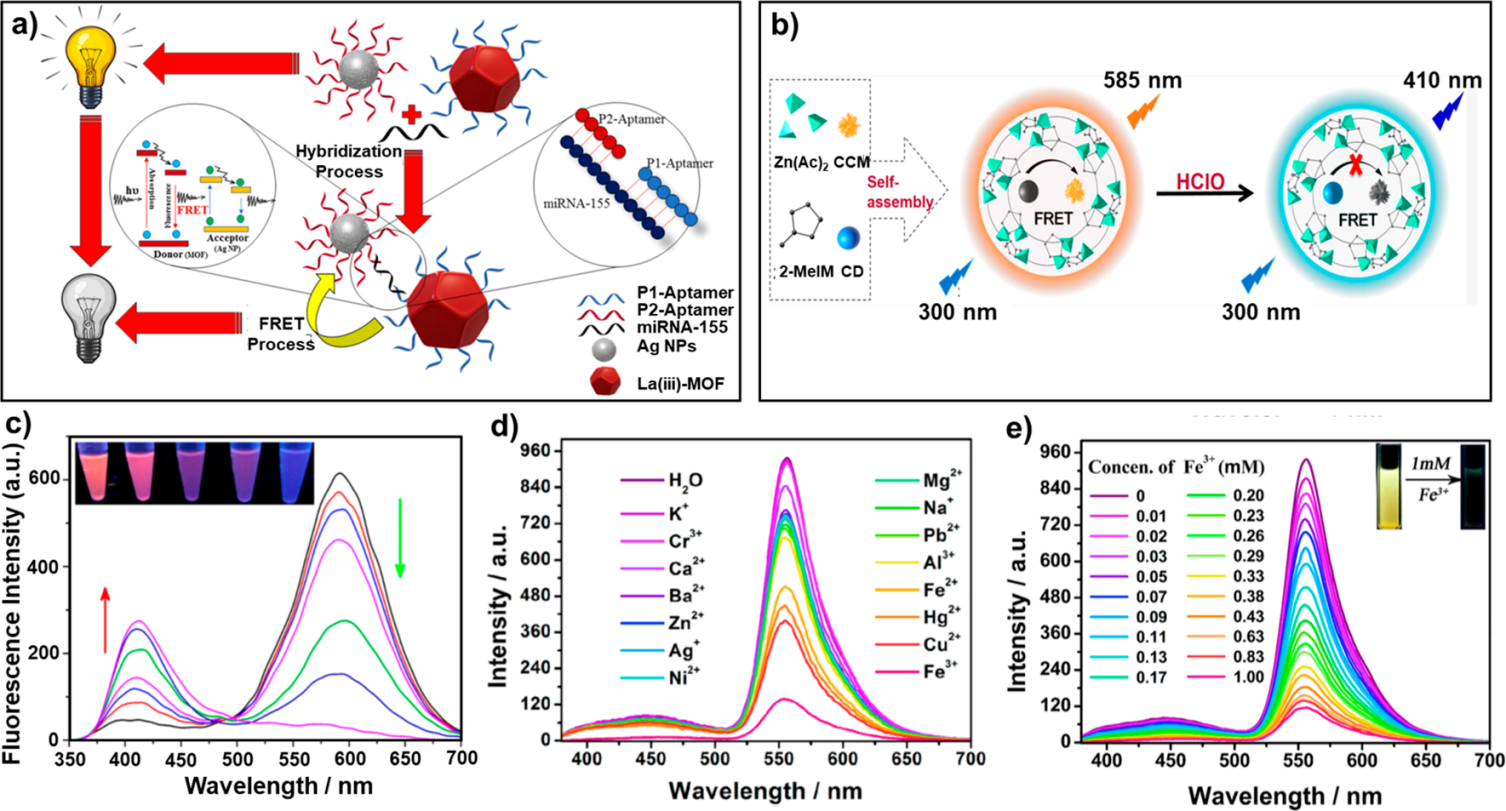
(a) Schematic diagram of photoluminescence quenching-based detection of miRNA-155 as a cancer biomarker. Reprinted with permission from ref (38). Copyright 2020 American Chemical Society. (b) Self-assembled FRET nanoprobe (CD/CCM@ZIF-8) for ratiometric sensing of hypochlorous acid, (c) emission spectra of CD/CCM@ZIF-8 after adding HClO (from 0 to 90 μM); inset is the corresponding fluorescent images under a UV lamp. Reprinted with permission from ref (156). Copyright 2020 American Chemical Society. (d) Fluorescence spectra of the EY@Zr-MOF in aqueous solutions with different metal ions (1 mM) upon 365 nm excitation, (e) concentration-dependent fluorescence spectra upon the different contents of Fe^3+^ in aqueous solutions; inset is the corresponding fluorescent images under a UV lamp. Reprinted with permission from ref (157). Copyright 2019 American Chemical Society.

Mirzaee’s group developed an ultrasensitive method for the detection of cancer biomarker miRNAs. They fabricated a “sandwich” biosensor through an oligonucleotide hybridization strategy. Once the miRNA integrated with the sensor, FRET from luminescent Ln^3+^-MOF to Ag nanoparticles was triggered, and the luminescence of the La(III)-MOF was quenched. The detection limit of this “turn-off” fluorescent biosensor is 0.04 ppb (ng mL^–1^), which provides a highly promising method for the diagnosis of lung and breast cancers (Figure 11a).^38^ Huang and co-workers reported a composite ratiometric sensor by entrapping carbon dot (CD) and curcumin (CCM) in ZIF-8 (CD/CCM@ZIF-8) for hypochlorous acid sensing. Because of the confinement effect of ZIF-8, the energy transfer from the carbon dots to curcumin is 68.7%, and the energy transfer process could be disrupted by hypochlorous acid, leading to the enhancement of the donor’s luminescence and the quenching of the acceptor’s luminescence (Figure 11b, c).^156^ By using a similar sensing mechanism, Xing and co-workers incorporated the fluorescent dye eosin Y into a UiO-type Zr-MOF (linker: 4,4′-stilbenedicarboxylic acid) to obtain a ratiometric sensor for the selective detection of Fe^3+^, Cr_2_O_7_^2–^, and 2-nitrophenol with excellent stability and high reversibility (Figure 11d, e).^157^

**Figure 12:**
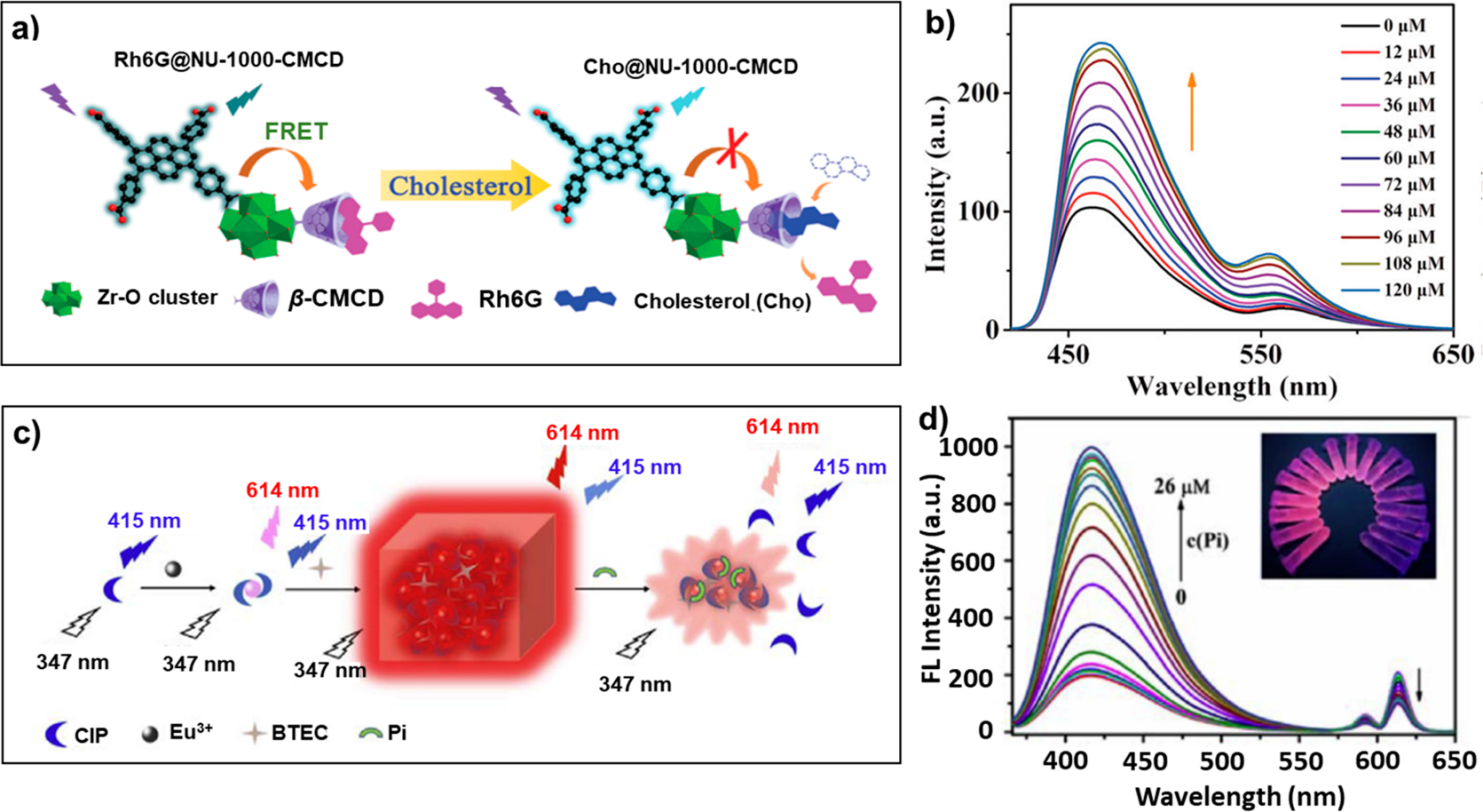
(a) Proposed sensing mechanism for cholesterol by using Rh6G@NU-1000-CMCD as the probe, and (b) fluorescence spectra of the sensing system at various cholesterol concentrations. Reprinted with permission from ref (146). Copyright 2019 The Royal Society of Chemistry. (c) Synthesis of Eu-MOFs for the ratiometric sensing of phosphate, (d) effect of the concentrations of phosphate on the fluorescence intensities of the MOFs (linker: pyromellitic acid); inset is the corresponding fluorescence photo under a 365 nm UV lamp. Reprinted with permission from ref (158). Copyright 2020 Elsevier.

Gu’s group reported a substitution-type LMOFs based sensor for selective cholesterol detection in blood serum. Carboxymethyl β-cyclodextrin was grafted onto the LMOF (NU-1000) with an inclusion site for cholesterol recognition. After having contact with the free cholesterol inside the serum, the encapsulated guest Rh6G was replaced by cholesterol to form a more stable inclusion complex. Thus, the quenched fluorescence of the LMOF was restored due to the disruption of FRET from the LMOF to Rh6G (rhodamine 6G) (Figure 12a).^146^ Tong and co-workers developed a lanthanide LMOF (BTEC-Eu-MOF, BTEC: pyromellitic acid) for ultrasensitive ratiometric sensing of phosphate (Pi) with a detection limit of 4.4 nM. An enhanced emission from the Eu^3+^ was achieved because of the efficient energy transfer from ciprofloxacin (CIP). After adding Pi to the solution of BTEC-Eu-MOF, CIP was released rapidly, and the energy transfer between CIP and Eu^3+^ was disrupted, leading to the recovery of the blue emission of CIP (Figure 12b).^158^ Ma and co-workers developed a nanoscale lanthanide LMOF (linker: benzene-1,3,5-tricarboxylic acid) as a colorimetric luminescence sensor for dipicolinic acid (DPA) sensing. DPA was combined with the Tb^3+^ LMOF by replacing the coordinated water and enhancing the green fluorescence of the LMOF. This changed the luminescence color from red-orange to yellow-green (Figure 12c, d).^159^

## 4 Luminescent Metal–Organic Frameworks: Films and Mixed-Matrix Membranes (MMMs)

MOFs synthesized by traditional techniques are commonly fine powders or tiny particles with intrinsic non thermoplastic properties, brittleness, and insolubility. Thus, processing MOF nanocrystals into specific films that are robust and exhibit operational flexibility is highly desired. Such technology not only preserves the individual advantages of MOFs and polymer matrices but also overcomes their drawbacks.^160−164^

### 4.1 Fabrication of Luminescent Metal–Organic Frameworks -Films and -Mixed Matrix Membranes

**Figure 13:**
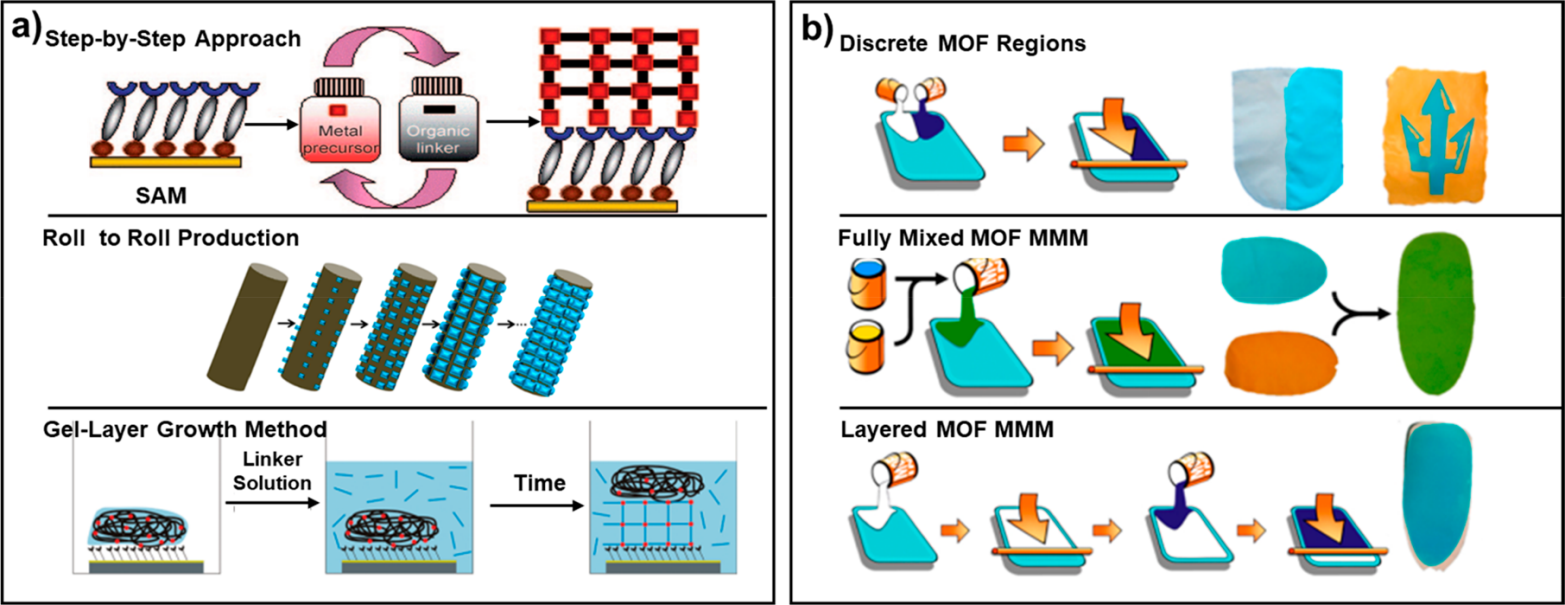
Preparation methods for (a) MOF-films and (b) MOF-based MMMs. Reprinted with permission from ref (16). Copyright 2012 American Chemical Society.

There are two main methods used for fabricating LMOF-films: direct growth of LMOF films and doping of LMOFs into polymer matrices.^65,165−167^ The direct fabrication of LMOF films includes surface-mounted MOFs (SURMOFs) and polycrystalline films.^71,168−170^ Polycrystalline films can be obtained through direct synthesis (in situ crystallization, fabrication at room temperature, dip coating in a mother solution and slow diffusion of reactants), seeded growth (MOF nanocrystals, non-MOF particles as seeds, and coordination polymers as seeds), electrochemical methods or stepwise dosing of reagents (Figure 13a).^16^ A useful alternative to shape LMOFs into desired configurations is construction of MMMs. MMMs are the kinds of composite membranes constructed by combining different kinds of hybrid materials into a polymer matrix. By using two or more materials with different luminescent and structural properties, these membranes demonstrate several unique advantages, such as easily fabricated shape and size, high chemical stability, good renewability, no invasive interference, and real-time detection of the analytes.^16^ With the increasing demand for new materials, methods for preparing MMMs by mixing two or more MOFs have been systematically studied. There are mainly three methods: (1) casting two MOF inks in discrete regions to obtain spatially separated MMMs; (2) combining two MOFs in one ink and then casting it on a substrate; (3) casting two MOFs ink layer by layer (LBL) to obtain discrete layered MMMs. The preparation methods of all the above-mentioned films have been described in detail in other reviews. Because these methods are beyond the scope of this review, please refer to the references for details (Figure 13b).^16,165^

### 4.2 Sensing Applications of Luminescent Metal–Organic Frameworks: Films and Mixed-Matrix Membranes

**Figure 14:**
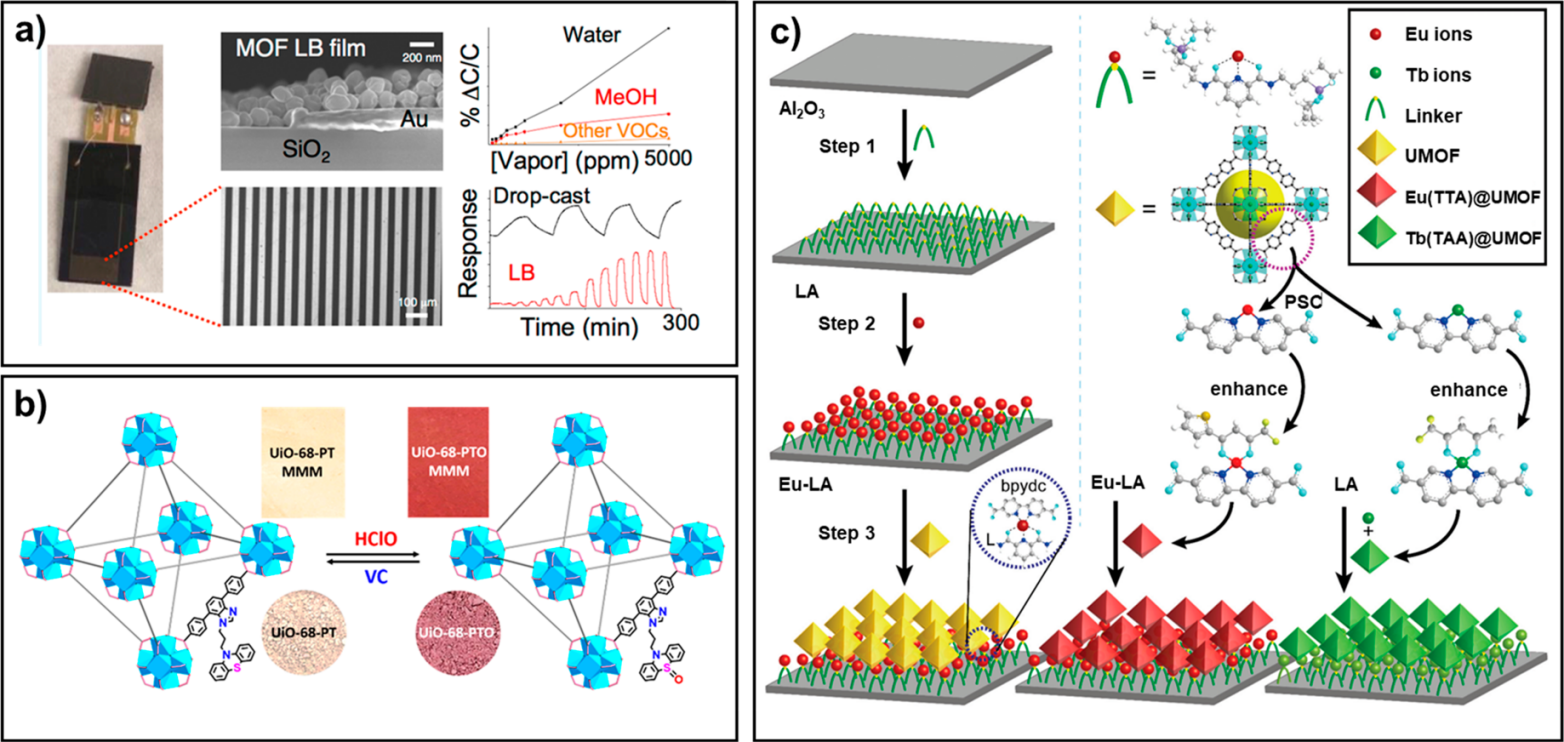
(a) Fabrication of the MOF LB film for the sensitive and selective sensing of water. Reprinted with permission from ref (25). Copyright 2020 American Chemical Society. (b) UiO-68-PT MOF-based MMMs for the sensing of HClO in aqueous solutions. Reprinted with permission from ref (171). Copyright 2019 American Chemical Society. (c) Fabrication process of lanthanide-functionalized UiO-67 films. Reprinted with permission from ref (172). Copyright 2020 The Royal Society of Chemistry.

Gascoń’s group reported a MIL-96 (metal: Al, linker: 1,3,5-benzenetricarboxylic acid) MOF thin-film-based probe for the selective detection of methanol and humidity. The thin films were fabricated via the LB (Langmuir–Blodgett) method on interdigitated electrode (IDE) chips. Presorption studies demonstrated that MIL-96(Al) presented a high affinity toward water and methanol, among other organic vapors. After depositing the MIL-96(Al) particles onto IDE ships, the selectivity toward methanol and water was achieved with short response/recovery times. Once the thin selective layer of Parylene C was deposited on top of the MOF LB films, the water selectivity and sensitivity were greatly enhanced while those of methanol showed a huge decrease (Figure 14a).^25^ Dong and co-workers developed a UiO-68-PT MOF- MMMs for the detection of HClO in water. Because of the reversible redox characteristics of the linker triggered by HClO and vitamin C, UiO-68-PT MOF exhibited high sensitivity toward HClO in water with both visual and fluorogenic enhancement. After mixing UiO-68-PT MOF into poly(vinyl alcohol), the fabricated MMMs still exhibited excellent HClO detection performance in aqueous solution (Figure 14b).^171^ Yan and co-workers reported a series of lanthanide LMOF thin films for the sensing of ammonia with low detection limits of 9 ppm. The linker of these LMOFs could combine with NH_3_ via hydrogen bonds, which increased the triplet energy of the linker and had adequate energy transfer toward Eu^3+^ ions. Thus, the Ln-LMOF films exhibited high selectivity for recognizing ammonia and were not interfered by other indoor pollutant gases (Figure 14c).^172,173^

## 5 Conclusions and Future Outlook

The photophysical and photochemical properties of LMOFs can be well modified by substituting metal nodes/organic linkers, changing their connectivity, and performing postsynthetic functionalization. More specifically, the coordination of organic linkers to metal nodes allows a high degree of chromophore organization, which is crucial for the precise determination of the distances and angles between self-assembled organic linkers, and modeling of the short- and long-range energy transfer processes. It should be pointed out that energy transfer in MOFs prevents exciton quenching and subsequently enhances the emission intensity of the energy acceptor unit, allowing the rational design of sensors.

Since the light-harvesting, energy transfer, and sensing processes could take place in the porous crystalline framework “three-in-one” sensing platform, MOFs show extraordinary sensing capabilities through proper structural design. In addition, the diversity of the MOF structures allows for the integration of light-harvesting units as either framework building blocks or guests. Consequently, the directional energy transfer between different frameworks functionalities (including ligand-to-ligand, metal-to-metal, metal-to-ligand (or ligand-to-metal), and guest-MOF energy transfer) could occur in a MOF matrix, which provides excellent sensing modes that could highly improve the sensing performance. Moreover, integrating external stimulus-responsive materials inside a MOF matrix to engineer stimulus-responsive sensors is another research hotspot associated with MOFs for sensing advancement.

Because of the drawbacks of MOF nanocrystals such as the brittleness, insolubility, difficulty in molding, and low compatibility with other materials, processing MOF nanocrystals into specific polymer matrices that are robust and exhibit operational flexibility is highly desired. Such technology not only preserves the individual advantages of MOFs and overcomes the drawbacks of MOF nanocrystals, but also expands the sensing applications to new directions. However, the research on MOFs-films or MMMs based sensors, especially the thin-films sensors related to energy transfer, is still in the preliminary stage. As a result, developing sensors based on ET-LMOFs films, and MMMs is highly demanded and should be one of the meaningful research directions because of their convenience and stability in practical applications.

In summary, the energy transfer phenomenon in LMOFs could highly improve their sensing capability. The amplified physical and chemical performance in the emissive centers by energy transfer processes and structural modifications of the LMOF matrix has made them excellent candidates for designing sensors with high sensitivity and selectivity.
